# Exploring deep residual network based features for automatic schizophrenia detection from EEG

**DOI:** 10.1007/s13246-023-01225-8

**Published:** 2023-03-22

**Authors:** Siuly Siuly, Yanhui Guo, Omer Faruk Alcin, Yan Li, Peng Wen, Hua Wang

**Affiliations:** 1 Institute for Sustainable Industries & Liveable Cities, Victoria University, Melbourne, Australia; 2 Centre for Health Research, University of Southern Queensland, Toowoomba, Australia; 3 Department of Computer Science, University of Illinois at Springfield, Springfield, IL 62703 USA; 4 Department of Electrical-Electronics Engineering, Faculty of Engineering and Natural Sciences, Malatya Turgut Ozal University, Malatya, Turkey; 5 School of Mathematics, Physics and Computing, University of Southern Queensland, Toowoomba, Australia; 6 School of Engineering, University of Southern Queensland, Toowoomba, Australia

**Keywords:** Electroencephalogram (EEG) signal, Schizophrenia detection, Deep residual network, Feature extraction, Classification

## Abstract

Schizophrenia is a severe mental illness which can cause lifelong disability. Most recent studies on the Electroencephalogram (EEG)-based diagnosis of schizophrenia rely on bespoke/hand-crafted feature extraction techniques. Traditional manual feature extraction methods are time-consuming, imprecise, and have a limited ability to balance accuracy and efficiency. Addressing this issue, this study introduces a deep residual network (deep ResNet) based feature extraction design that can automatically extract representative features from EEG signal data for identifying schizophrenia. This proposed method consists of three stages: signal pre-processing by average filtering method, extraction of hidden patterns of EEG signals by deep ResNet, and classification of schizophrenia by softmax layer. To assess the performance of the obtained deep features, ResNet softmax classifier and also several machine learning (ML) techniques are applied on the same feature set. The experimental results for a Kaggle schizophrenia EEG dataset show that the deep features with support vector machine classifier could achieve the highest performances (99.23% accuracy) compared to the ResNet classifier. Furthermore, the proposed model performs better than the existing approaches. The findings suggest that our proposed strategy has capability to discover important biomarkers for automatic diagnosis of schizophrenia from EEG, which will aid in the development of a computer assisted diagnostic system by specialists.

## Introduction

Schizophrenia is a complex and severe mental illness, which affects about 1 in 100 Australians (between 150,000 and 200,000) [1, 2] and approximately 20 million people worldwide [3]. People with schizophrenia mostly experience hallucinations, delusions, disorganized speech and behaviour, movement disorders, [3]. The schizophrenic patients face significant problems in their daily life living at diverse levels of social, cognitive, emotional, physiological, and psychological functions [4]. It was estimated that 20%–40% of schizophrenic patients attempt suicide at least one time, and 5%–10% of them successfully carried out suicide [5]. According to the World Health Organization (WHO) report, schizophrenia is linked with considerable disability that lead to high health care expense globally [3]. There is no cure for schizophrenia, but it can be treated and managed using long-term medication that causes an excessive load on the health care system and patients’ family [6]. Thus, accurate and early diagnosis is needed for better treatment that can significantly improve patients’ survival and quality of life and reduce health care costs.

There are several techniques to diagnose schizophrenia such as interview method, computed tomography (CT), magnetic resonance imaging (MRI), electroencephalography (EEG), etc. Among them, the interview diagnosis method is not reliable because it is a manual process which is laborious, onerous, subject to error, and unfairness. The neuroimaging techniques (e.g. MRI, CT) are expensive and require additional recording and computational time as compared to the EEG technique [7, 8, 9, 10, 11]. Currently, EEG is emerged as the reference standard for diagnosis of schizophrenia owing to its high temporal resolution, non-invasiveness, and relatively low financial cost compared to other tests [12]. The EEG technique records electrical activity of the brain through electrodes placed on scalp [4]. Produced EEG recordings convey information about the state of brain. Different brain-related disorders generate different patterns of EEG signals. EEG recording contains huge volumes of dynamic data to study brain function. Usually, this large amount of data is assessed by visual inspection, which takes long time, subject to human error, and reduces decision-making reliability [13, 14]. As yet, there is no reliable way of identifying schizophrenia from EEG data automatically, rapidly, and accurately. Therefore, the motivation of this study is to develop an automatic schizophrenia detection scheme using EEG signal data.

In recent years, many research studies have been performed to acquire informative features from EEG signals for the detection of schizophrenia [15, 16, 17, 18, 19, 20, 21, 22, 23, 24, 25, 26, 27, 28]. These studies reported various algorithms based on various features to characterize the.

EEG-based brain region. Some of the past works in the area of schizophrenia detection are reported in as shown in Table 1. In literature, it is observed that most of the research work in the schizophrenia detection from EEG signals are based on bespoke/hand-crafted feature extraction methods such as wavelet transformation, fast Fourier transformation, power spectral density, spatial pattern of network, Kolmogorov complexity, entropy, etc. [15, 16, 17, 18, 19, 20, 21, 22, 23] where the methods are manually chosen based on the researcher’s expertise. The hand-crafted feature extraction methods cannot form intellectual high levels of representations of EEG signals to discover deep concealed characteristics from data that can achieve better performance. Sometimes it is difficult to choose effective feature extraction methods for different structures of EEG data and this is labor-intensive and time-consuming. Moreover, the methods underperform when the large datasets are used. In literature, very few research works have been performed on deep learning (DL) for detection of schizophrenia in EEG (e.g. we found few works [24, 26, 28]) but the methods are still limited in their ability to balance the efficiency and accuracy of schizophrenia detection. In addition, we also explored some recent works of schizophrenia detection that used Magnetic resonance images (MRI) data [29, 30, 31, 32, 33, 34] as reported in Table 1. Their performances weren’t really up to par.

**Table 1:** Some recent research works to identify schizophrenia with EEG data and MRI data (2019–2022)

Studies	Data set structure	Length of signal	Methods		Best reported performance (%)
			For feature extraction	For classification	
Miras et al. [15] 2022	EEG data [15]; 20 healthy control (HC) subjects and 11 subjects suffering from schizophrenia	3 min	HFD, ApEn, SamEn, CD, LZC, MIMR, DFA, HA, HM and HC, DP, TP, AP and BP and TE, LLE, DET	*k*-nearest neighbors algorithm (kNN)	Accuracy: 87.0 Sensitivity: 82.0 Specificity: 90.0
H. Akbari et al. [16] 2021	EEG data[17, 18]; 14 schizophrenias, 14 HCs	–	Graphical feature extraction	kNN	Accuracy: 94.80 Sensitivity:94.30 Specificity: 95.20
K. Kim et al. [19] 2021	EEG data [17, 18]; 14 schizophrenias, 14 HCs	5 s	Microstate features; statistical, frequency, and time domain features; t-test; recursive feature elimination;	Support vector machine (SVM)	Accuracy: 75.64 Sensitivity: 71.93 Specificity:75.50
R. Buettner et al. [20] 2020	EEG data [17, 18]; 14 schizophrenias, 14 HCs;	1 min	Spectral analysis-based feature extraction	Random forest (RF)	Accuracy: 96.77 Sensitivity: NA Specificity: NA
P.T. Krishnan et al. [21] 2020	EEG data [17, 18]; 14 schizophrenias, 14 HCs	2 s	Multivariate empirical model decomposition and entropy computation	SVM	Accuracy: 93.0 Sensitivity: 94.0 Specificity:92.0
V. Jahmunah et al. [22] 2019	EEG data [17, 18]; 14 schizophrenias, 14 HCs	25 s	Nonlinear statistical moment-based feature extraction	SVM	Accuracy: 92.91 Sensitivity: 93.45 Specificity: 92.24
Li et al. [23] 2019	EEG data [23]; 48 right-handed subjects, 23 SZs and 25 HCs	1 s	Spatial pattern of network (SPN) features	SVM	Accuracy: 90.48 Sensitivity: 89.47 Specificity: 91.30
Ko and Yang [24] 2022	EEG data [25]; 49 schizophrenic patients and 32 healthy subjects	3 s	Gramian Angular Field (GAF) for converting EEG signals to images	Convolutional Neural Network (CNN): VGGNet	Accuracy: 93.20
Z. Aslan and M. Akin [26] 2020	Two EEG data sets: Data set 1: 45 schizophrenias, 39 HCs [27]; Data set 2: 14 schizophrenias, 14 HCs [17, 18];	5 s	EEG signals were converted into Spectrogram images	VGG16 deep network	For data set 1: Accuracy: 95.0 Sensitivity: NA Specificity: NA For data set 2: Accuracy: 97.0 Sensitivity: NA Specificity: NA
Phang et al. [28] 2019	EEG data [27]; 45 schizophrenias, 39 HCs	5 s	Deep belief network (DBN)	Softmax	Accuracy: 95.0
Levman et all [29] 2022	Magnetic resonance images (MRI) data [30]; 99 schizophrenia patients, and 75 healthy control subject	–	correlation analysis	SVM	Accuracy: 92.0 Sensitivity: - Specificity: 74.0
Ghanbari et al. [31] 2021	MRI data [30]; 72 subjects of schizophrenia patients and 74 subjects of healthy controls	–	Three-dimensional CNN and long short-term memory recurrent network (LSTM)	Softmax	Accuracy: 92.32 Sensitivity: 93.91 Specificity: 88.7
Zheng et al. [32] 2021	MRI data [30]; 98 schizophrenia patients, 102 healthy subjects	–	CNN: VGG16	Sofmax	Accuracy: 85.27 Sensitivity: 87.48 Specificity: -
Oh et al. [33] 2020	Five public MRI data sets [32]; 449 schizophrenia patients, 424 normal subjects	–	Deep neural networks	Softmax	Accuracy: 97.00 Sensitivity: 96.00 Specificity: 96.00
Lei et al. [34] 2019	MRI data [34]; combination of five data sets 295 schizophrenias, 452 HCs;	–	Gray matter, white matter, low-frequency fluctuation, regional homogeneity, structural covariance matrices, and functional connectivity matrices	SVM	Average Accuracy: 87.59 Sensitivity: 81.02 Specificity: 94.17

*HFD* Higuchi fractal dimension, *ApEn, SamEn* Approximate and sample entropy, *CD* Correlation dimension, *LZC* Lempel–Ziv complexity, *MIMR* Mutual information of multiple rhythms, *DFA* Detrended fluctuation analysis, *HA, HM and HC* Hjorth activity, mobility and complexity, *DP, TP, AP and BP* Power of delta, theta, alpha and beta bands, and *TE* total energy, *LLE* Largest Lyapunov exponent, *DET* Recurrence quantification analysis

*NA*  not available, ‘ - ‘  not reported

Hence, this study introduces a deep learning-based feature extraction method employing a deep residual network (deep ResNet) model for the detection of schizophrenia using EEG signals data. The main advantage of the DL method is that there is no need to manually extract features from the signals. The network learns to extract features while training which can help to enhance the speed and effectiveness of supervised learning. A deep ResNet is a special form of neural network that helps to handle more advanced DL tasks and models. In neural network architectures, the deep ResNet model has capability to produce better performance through a network with many layers (optimum number of layers) overcoming the “vanishing gradient” problem. The proposed framework is designed with three phases: signal pre-processing, extraction of hidden patterns from EEG signals, and classification of schizophrenia. This study employs an average filtering method to pre-process the raw EEG data. The deep ResNet framework is developed to automatically extract concealed significant features from EEG signals to identify schizophrenia patients from normal control subjects. The obtained deep ResNet features are used as input to the softmax classifier. In order to further assess the performance, the deep ResNet feature set is also fed into several ML techniques. A detailed explanation of the mentioned methods is provided in Section II (A) with the motivations that these methods are considered in this study.

The main contributions of this work are summarized as follows: (1) for the first time, we introduce a deep ResNet based DL framework, for mining deeper features from EEG to automatically identify schizophrenia from normal control subjects; (2) we discover a sustainable classifier for the attained deep feature set from the DL and ML environment; (3) we investigate the performance of the deep ResNet feature set with several ML methods for detection of schizophrenia; (4) we enhance the identification performances than the state-of-arts methods.

## Methodology

**Fig.1:**
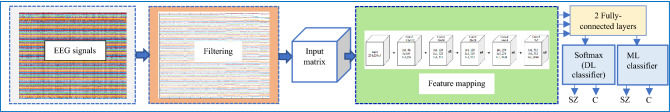
The overall architecture of the proposed deep ResNet based framework for detection of schizophrenia from EEG signals. *SZ Schizophrenia, *C Normal Control, *DL Deep learning, *ML Machine learning

The aim of this study is to develop a deep RestNet based DL framework that can use EEG signal data to automatically and efficiently identify schizophrenia patients from normal control subjects improving the classification performance. The architecture of the proposed deep RestNet based framework for the diagnosis of schizophrenia is presented in Fig. 1 and the configurations of the proposed networks are outlined in Table 2. A brief description of the proposed framework is provided below.

**Table 2:** Structure of the proposed deep resnet model used in this study

Layer name	Output size	50-layer
Conv1	112 × 112	7 × 7, 64, stride 2
Conv2_x	56 × 56	3 × 3 max pool, stride 2
		$$\left[\begin{array}{c}1\times \mathrm{1,64}\\ 3\times \mathrm{3,64}\\ 1\times \mathrm{1,256}\end{array}\right]\times 3$$
Conv3_x	28 × 28	$$\left[\begin{array}{c}1\times \mathrm{1,128}\\ 3\times \mathrm{3,128}\\ 1\times \mathrm{1,512}\end{array}\right]\times 4$$
Conv4_x	14 × 14	$$\left[\begin{array}{c}1\times \mathrm{1,256}\\ 3\times \mathrm{3,256}\\ 1\times \mathrm{1,1024}\end{array}\right]\times 6$$
Conv5_x	7 × 7	$$\left[\begin{array}{c}1\times \mathrm{1,512}\\ 3\times \mathrm{3,512}\\ 1\times \mathrm{1,2048}\end{array}\right]\times 3$$
	1 × 1	Average pool, 2-d FC, softmax
FLOPs		3.8 × 10^9^

### Deep learning (DL) framework

The key important characteristic of the DL model is to automatically extract effective features, which has benefit for large volume data. The proposed DL framework consists of three steps as shown in Fig. 1: (1) signal pre-processing; (2) extraction of hidden patterns from EEG signals and (3) classification of schizophrenia. Step 1 involves with the signal pre-processing where the noise and artifacts of EEG signals are removed employing an average filtering method. Step 2 comprises the feature extraction where the hidden concealed patterns are extracted from denoised signals using the deep ResNet model to enable efficient detection of schizophrenia. Finally, step 3 performs the classification task where the extracted deep features are fed to softmax classifier to classify schizophrenia from normal control subjects. Also, the same feature set are fed as input to several ML classification methods for classifying schizophrenia classification. The detail description of the three steps are provided below:

#### Step 1. Signal pre-processing

EEG data are intrinsically noisy and often disturbed by artifacts. The noise and artifacts might bias the analysis of the signals that lead to incorrect conclusions. The key aim of pre-processing is to minimise or, eventually, eliminate noise and artifacts (e.g. outliers) from the signals. This process standardizes the data so that they can be easily used to create an appropriate model. This study employs an average filter for reducing and removing noise from EEG data. In the average filtering process, we smooth each signal (Sg) with a kernel size, *KLen* = *12* using Eq. (1), and the smoothed signal is sampled as $\widehat{\mathrm{Sg}}$ with an interval length *IntLen* = *KLen* = *12* using Eq. (2). To create

1 $$\overline{\mathrm{Sg} }(\mathrm{t})=\frac{1}{KLen}\sum_{i=t-\frac{KLen}{2}}^{t+\frac{KLen}{2}}Sg\left(i\right)$$

2 $$\widehat{{{\text{Sg}}}}\left( {\text{t}} \right) = \mathop \sum \limits_{{i = 0}}^{L} \delta \left( {i - t \cdot IntLen} \right) \cdot \overline{{{\text{Sg}}}} \left( i \right)$$

the final matrix, which has a size of about 200, the values of KLen and IntLen are determined by trial and error. The raw signal data then serves as an input matrix for feature extraction in the deep ResNet model, which is discussed in the next step. This filtering was chosen for this study because it is straightforward, intuitive, and simple to use in order to smooth the signals and reduce the amount of intensity variation that may remove unrepresentative (unwanted) information from the signals.

#### Step 2. Extraction of hidden patterns of EEG signals by deep resnet model

This section aims to extract salient important features robustly and automatically from EEG data that enable efficient detection of schizophrenia. Feature extraction is the most important stage of a classification task. Transforming denoised EEG signals by an ideally small number of relevant values that describe task-related information is called ‘feature’*.* Traditional hand-crafted feature extraction methods choose features manually from data which is tedious and unsophisticated. The DL based methods extract meaningful features in an automatic way directly from the EEG data using less time that empower to generate better performance of recognition compared to the traditional methods. This study introduces a deep ResNet architecture to extract significant representative features from the denoised EEG signals. As per our knowledge, for the first time, the deep ResNet is employed in this study for the detection of schizophrenia from EEG signals. Previously, this method was employed on image recognition [34, 35, 36, 37, 38, 39] and epilepsy detection [40] but was not applied in the schizophrenia detection yet.

The motivation of using the deep ResNet model in this study is that this method is developed as a family member of extremely deep architectures showing convincing accuracy and nice convergence behavior in considerably increased depth. The deep ResNets built up with an advanced residual unit and full pre-activation [35], where identity mappings are based on skip layer styles to ease the vanishing/exploding gradient issues [41]. The deep ResNet makes better networks as easy as adding more layers onto it for having representative hierarchical discriminative features from data. In the deep ResNet structure, the feature of any deeper unit is characterized as the feature of any shallower unit plus the summation of the preceding residual responses. This characteristic helps to substantially improve performance on ultra-deep networks with more than 1000 layers compared to earlier residual methods [42]. The deep ResNet has capability to optimize and gain accuracy from considerably increased depth [35].

**Fig. 2:**
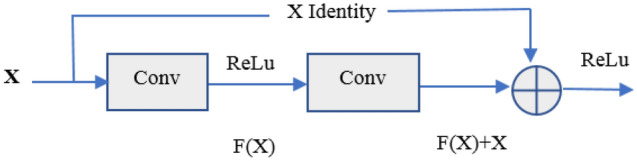
Residual learning block

The ResNet is constructed based on feedforward deep neural networks developed in [35], where each layer uses different operations to extract information from the previous layers, and also has a unique residential operation in the network. The networks utilize skip connections, or shortcuts to jump over some layers. A detailed description of the deep ResNet architecture is available in refs [35, 43]. Typically, ResNet involves with convolutional (Conv) layers, rectified linear units (ReLu) layers, batch normalization layers, and lay skips which is demonstrated in Fig. 2.

Figure 2 illustrates an example of the residual learning module. In this figure, ‘X’ is the mapping of input, and F(X) is a residual function. As depicted in Fig. 2, after two branches (mapping ‘X’ of input and residual function F(X)) are combined, they are exposed to nonlinear transformation (ReLu activation function) to create the entire residual learning module. As shown in Fig. 2, the input is X and a shortcut is added after X, and the output of the block is superimposed upon the input. Therefore, the output of the block becomes F(X) + X, and the network weight parameters need to learn is F(X). In Fig. 2, the most useful information to realize is the ‘skip connection’ in identity mapping. This identity mapping does not have any parameters and is just there to add the output from the previous layer to the layer ahead [35]. In the proposed model, residual blocks are used in the whole network as the basic units of deep ResNet which is defined as below [44]:

3 $${\text{Y = F}}\left( {{\text{X,}}\left\{ {{\text{W}}_{{\text{i}}} } \right\}} \right){\text{ + X}}$$

Here X denote input vector and Y denote output vector of the given layers. The function F(X, {W_i_}) symbolizes the residual mapping to be learned. In Eq. (3), the dimensions of X and F should be identical.

In this study, we design the structure of the deep ResNet model for implementation reported in Table 2. This table shows a structure of the network setting how it is implemented with large numbers of parameters on the data in this study. This study extracts deep features from five convolutional layer with several kernels, and then the activation function works on the convoluted results to generate the feature maps. The polling layer acquires the feature from the previous layers. The average polling layer uses the average value from each cluster of neurons of the previous layer. Finally, 2 fully connected layers are used, and their outputs are fed to the softmax function for classification task.

#### Step 3. Classification of schizophrenia

This stage aims to perform the schizophrenia classification task using the obtained deep feature set. As shown in Fig. 1, after five convolutional layers and one average pooling layer, there are two fully connected layers which are the final layer of the deep ResNets model that produces outputs, a probability distribution over all possible categories for the given input. In the final layer, this study uses a softmax function where there are an output ranges from 0 to 1 per class for classification. These outputs refer to the predicted probability of the signal belonging to a certain class, where higher probabilities indicate as the more confident that the input belongs to that class [45]. As seen in Fig. 1, in the fully connected layer, the output vector of the former layer is used as input to the softmax function. The below formula is applied to generate the probabilities of estimated results for the output of the softmax function:

4 $$P(y=1|x,w,b)=\frac{\mathit{exp}(w\cdot x+b)}{1+\mathit{exp}(w\cdot x+b)}$$

5 $$P(y=0|x,w,b)=\frac{1}{1+\mathit{exp}(w\cdot x+b)}$$

where $y$ refers to target class, x refers to a feature vector, w refers to the weight parameter, and $b$ refers to a bias term.

This study uses a binary cross-entropy function as the loss function which calculates the binary cross-entropy (BCE) between estimates and targets [46], which is given by:

6 $$BCEL=-\frac{1}{{N}_{0}}{\sum }_{i=1}^{{N}_{0}}\left[{t}_{i}\mathit{log}({p}_{i})+(1-{t}_{i})\mathit{log}(1-{p}_{i})\right]$$

where ${N}_{0}$ indicate the total number of training samples, ${p}_{i}$ indicate the estimated result of each sample, and ${t}_{i}$ indicate the target class in $\left\{\mathrm{0,1}\right\}$, where 0 refers to normal control and 1 refers to schizophrenia patient.

In order to investigate the efficacy of the obtained deep ResNet feature set, this study also used the same feature set to each of five ML classification methods: SVM, *k*-NN, DT, LD, and NB. The reason for the choice of these classifiers for this study is because those classifiers are straightforward and very effective in implementation. In addition, those ML classifiers have powerful and speediest learning capability to investigate all the training inputs in the classification process [12, 13, 14, 47, 48].

#### Step 4. Performance evaluation

In this step, the performances of the proposed model is evaluated using practically standard measurements such as accuracy, sensitivity, specificity, precision (positive predictive value), false positive rate (false alarm rate), false negative rate (miss rate), F1 score and operating characteristic curve (ROC) area [12, 49, 50, 51, 52].

## Results

Firstly, this section presents a brief description of the data that is used in this study. Afterward, the design of the method implementation is described including the parameter setting information of the proposed the deep ResNet model. Then, feature extraction process is discussed at the end of this section. Afterward, the obtained results are discussed in Section IV.

### Data acquisition

The study used an EEG signal database from Kaggle data source. That database consists of 81 subjects EEG data where 49 subjects are schizophrenia patients, and 32 subjects are normal control persons. Out of 81 subjects, 14 subjects are female, and 67 subjects are male. The average age is 39 and the average education is 14.5 years. EEG data were collected from total of 70 channels including 64 channels on the scalp and 6 channels around the eyes and nose. The data were continuously digitized at 1024 Hz and referenced off-line to averaged earlobe electrodes. The length of each signal was 3 s. EEG was measured 100 times each under these three conditions. (1) Subject pressed a button to generate the tone. (2) Subject passively listened to the same tone. (3) Subject pressed a button without generating the tone. In this study, we test only condition one to classify SZ patients and HC subjects because the HC subjects generate a press button tone while the SZ patients did not [53]. A details description of this dataset is available in reference [25] and [53].

### Implementation

In this study, all experiments were performed in MATLAB (2018b) on a PC with a Six-Core Intel i7 processor and 32 GB of memory. The server was equipped with an NVIDIA RTX 2060 GPU with 6 GB of memory. The deep ResNet model was run in MATLAB Deep Learning Toolbox. This study implemented the proposed method on a schizophrenia EEG data. As mentioned before, this data contains EEG recordings of 81 subjects including 32 normal control subjects and 49 schizophrenia patients. The EEG recordings of normal control subjects contains 3108 trails, 3072 samples per trial and 70 channels, and the recording of schizophrenia subjects contains 4608 trails, 3072 samples per trial and 70 channels. Now we are going to show an example of how our data are processed with the proposed model in this study. Here we provide an example of data processing and transforming for subject 1.

**Fig. 3:**
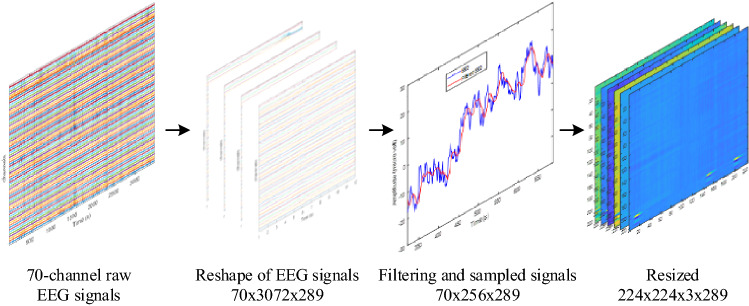
**a** 70 × 256 size image; **b** 224 × 224 resized image of (a); **c** 224 × 224x3 resized image of (b)

**Fig. 4:**
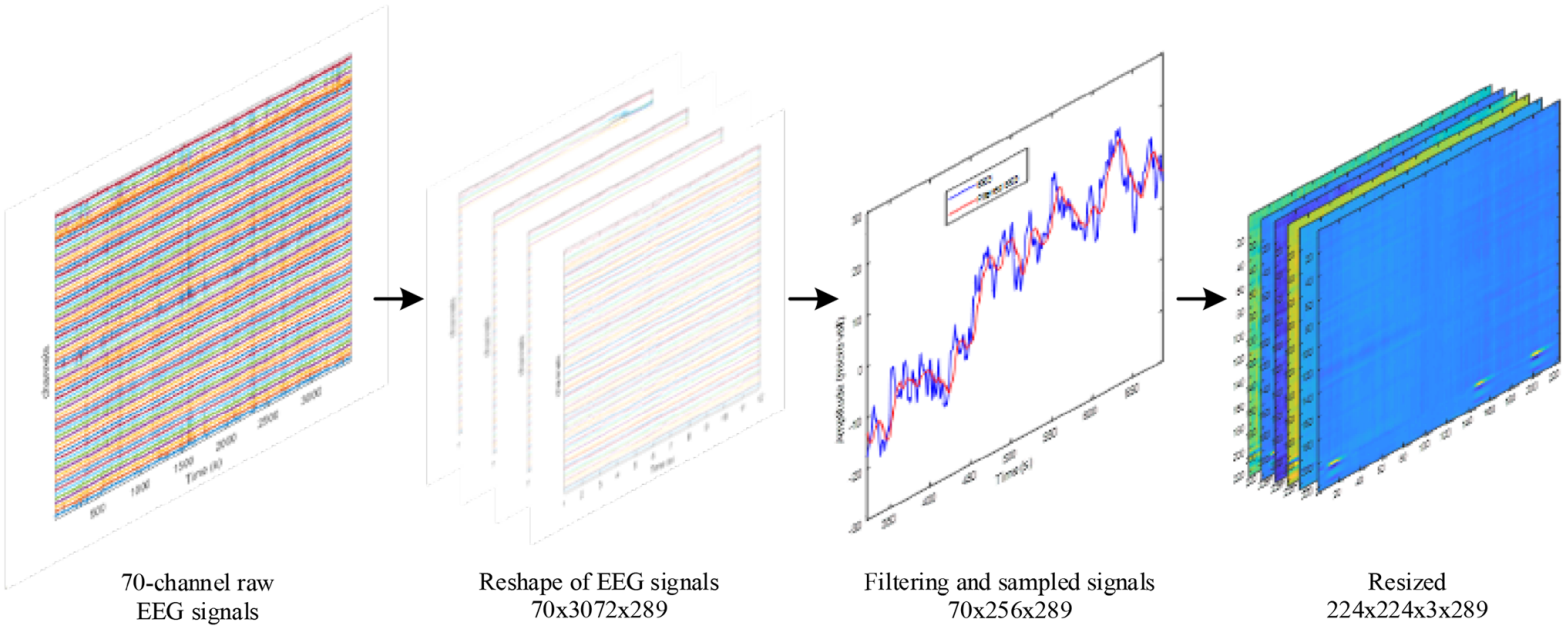
An example of signal data transformation to image data for subject 1

For example, Subject 1 is made up of 887,808 × 70 data (samples x channels), which was transformed into a matrix with the dimensions 70 × 3072x289 (channels x samples x images). By using 887,808/3072, 289 was achieved. After applying average filter, the raw signal data matrix was converted to a matrix with a size of 70 × 256 × 289. Afterward, the 70 × 256x289 matrix was resized to 224 × 224x3 × 289 (height x width × 3 symbolize color layer x images) to be compatible with the deep ResNet input size for subject 1. An image sized 70 × 256 grey scale image is shown in Fig. 3a and this image is resized to 224 × 224 with grey-scale format as shown in Fig. 3b. As the grey scale format is not compatible for ResNet inputs, thus the grey-scale images are concatenated to 224 × 224 × 3 (height × width × color depth) as shown in Fig. 3c. Here color depth is generated by combining the same grey scale image three times. In this work, the “*imresize*” function was used to accomplish the resize operation in Matlab environment. The “*imresize*” function applies the nearest-neighbor, bilinear, or bicubic interpolation strategy to interpolation. Bicubic interpolation was utilised as hyper-parameters in the paper. Figure 4 shows an example for subject 1 how the signal data were transformed into image data for deep ResNet application.

Following a similar process, for a total of 81 subjects, the whole dataset was transformed into an image matrix with size 224 × 224x3 × 23,201 (height x width × 3 symbolize color layer x image samples). Then, this image data was divided into three parts: training, validation, and testing with ratios 70%, 10%, and 20%, respectively detail given below in Table 3. Table 3 provides the size of the three groups of data. In this study, the training dataset was used for learning process of the proposed model and the validation data set was regarded as a part of training set to tune the model. The validation set was used for tuning the parameters of a model and also for avoiding overfitting. Generally, the validation data set helps provide an unbiased evaluation of the model’s fitness. The testing dataset was used for performance evaluation.

**Table 3:** Sizes of three groups data sets points

Sample		Training set	Validation set	Testing set
Dataset	X	224 × 224x3 × 16,240	224 × 224x3 × 2320	224 × 224x3 × 4641
	Y	16,240 × 1	2320 × 1	4641 × 1

X sample points, Y data labels

**Fig. 5:**
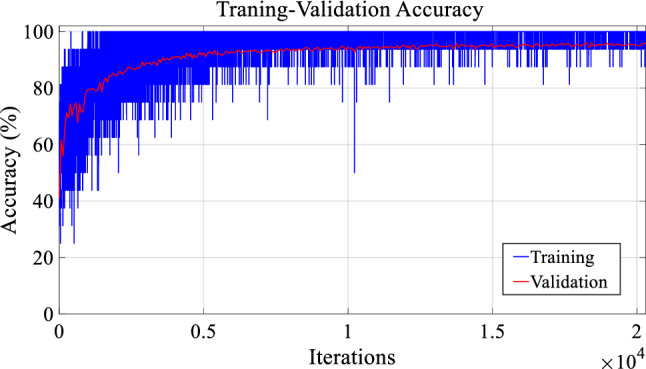
An example of network training in different iterations

To demonstrate the superiority of the proposed model, this study conducted extensive experiments and investigate the performance of the deep ResNet method with several ML methods. The hyper-parameters of the deep ResNet structure is provided in Table 2. All of the factors were fine-tuned based on the training set that provides the optimal training accuracy. Figure 5 displays an example of training and validation accuracy for the deep ResNet classifier in different iterations. It is seen that during training, there was no significant improvement on validation accuracy in some iterations. In this study, we considered the learning rate as 0.0001 for the training stage, and took one sample each time as the batch number. We used the brute force technique for determining optimal number of filters and kernel sizes. In the DL environment, the softmax function was used in the fully-connected (FC) layer for classification.

As mentioned before, besides DL classifier, this study also uses five ML classifiers: SVM, *k*-NN, DT, LD and NB for classification purposes to compare the performances of the obtained deep features. In this study, we selected the linear kernel for SVM as the best kernel function. Because, after extensive experiments using several kernels such as linear, polynomial, radial basis kernel), we found that linear kernel suits better with the acquired feature set than other kernel functions. For *k*-NN, we considered ‘Euclidean distance’ for distance measure and used the *k* value *as* 1 after some experimental investigations. Since there is no standard rule for choosing k, in this study we used an empirical method with a range of k values from 1 to 20 and selected a suitable k value that provides the lowest error rate, as the optimal model is the one with the lowest error rate. For DT, LD, and NB classifiers, we considered the default parameter settings in MATLAB because we could not find any standard rule to choose the values of the parameters of those classifiers.

### Feature extraction

**Fig. 6:**
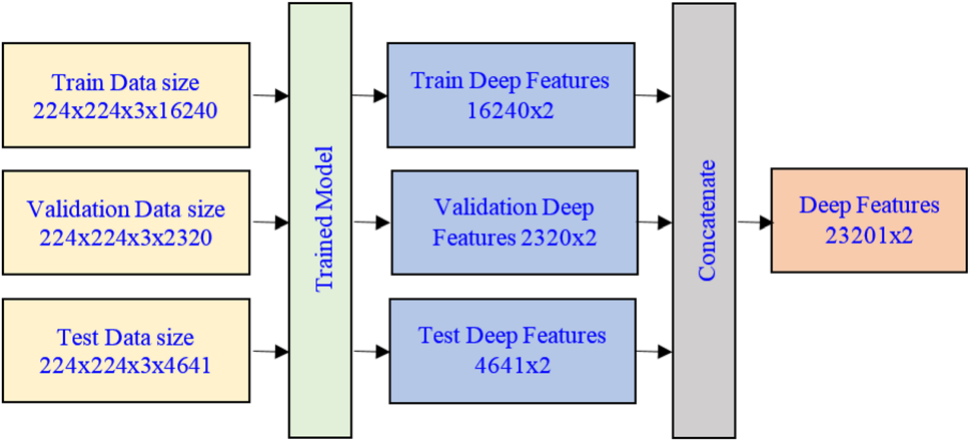
The feature extraction process by deep ResNet model

This section provides information on how EEG signal data are converted into a deep feature set by the deep ResNet model. Figure 5 presents a diagram of the data processing of how they are converted into the feature set. The sizes of the data are also indicated in this figure. As can be seen in Fig. 6, the proposed model generates two deep leaning features, and the size of the obtained deep feature set is 23201 × 2. To estimate the performance of the classification models, this study applied a 10 folds cross-validation technique on this obtained deep feature set. In 10 folds cross-validation, the original sample is randomly partitioned into 10 equal size fold. Of the 10 folds, a single fold is taken as the validation data for assessing the models, and the rest 9 folds are employed as the training data. The cross-validation process is then repeated 10 times, with each of the 10 folds used exactly once as the validation data. The average of the 10 folds’ results is considered as a separate evaluation for consideration.

**Fig. 7:**
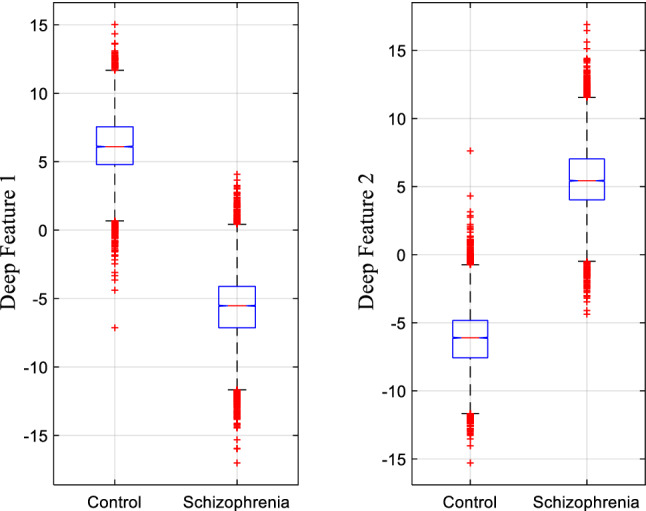
Boxplots for the deep ResNet features grouped by Control and Schizophrenia

Figure 7 presents a box plot showing the distribution of two obtained deep features: deep feature 1 and deep feature 2 in the Control and Schizophrenia category. It is apparent from Fig. 7 that both control and schizophrenia groups are showing same distribution pattern, which is symmetric, but a considerable variation is observed in the central value of both groups. The boxplot figures illustrate that features values between two groups values have significance difference that make easier to have efficient classification performance.

## Discussion

This section presents discussion for the obtained experimental results of the proposed schizophrenia detection scheme. The experimental results are obtained based on binary classification process (2 class classification). Here, the schizophrenia signals are considered as one class and the normal control signals are considered as another class e.g. classification of schizophrenia and control.

Table 4 presents the overall classification results for the obtained deep features with the softmax classifier of deep ResNet model (DL classifier) and also with the four ML classifiers: SVM, *k*-NN, DT, LD and NB, separately. In this table, the overall performances are reported in terms of accuracy, sensitive, specificity, precision and F1-score. It is observed from Table 4 that the proposed deep ResNet features deliver reasonably better performance results for each of the reported ML classifiers compared to the DL classifier. As can be seen from Table 4, in most of the cases, the highest classification performances are achieved for the SVM classifier among the reported classifiers, which are 99.23% of accuracy, 99.02% of specificity, 99.36% of precision and 99.36% of F1-score. On the other hand, the DL classifier yields the lowest performances, where accuracy, specificity, precision, and F1-score values are 97.48%, 97.90%, 98.58%, and 97.88%, respectively. The highest sensitivity (99.44%) is obtained by the LD classifier while the lowest sensitivity (97.19%) is attained by the DL classifier. Thus, the results in Table 4 demonstrate that the deep RestNet features work better with the ML classifiers compared to the DL classifier. The overall accuracy is increased by 1.75% for the ML-based classifier compared to the DL scheme.

**Table 4:** Classification performances for the obtained deep features with the various classifiers

Method	Accuracy (%)	Sensitivity (recall) (%)	Specificity (%)	Precision (%)	F1 score (%)
Deep ResNet	97.48	97.19	97.90	98.58	97.88
SVM	99.23	99.36	99.02	99.36	99.36
*k*-NN	98.88	99.08	98.58	99.06	99.07
DT	98.85	99.04	98.56	99.05	99.04
LD	99.20	99.44	98.84	99.24	99.34
NB	99.21	99.39	98.92	99.29	99.34

**Fig. 8:**
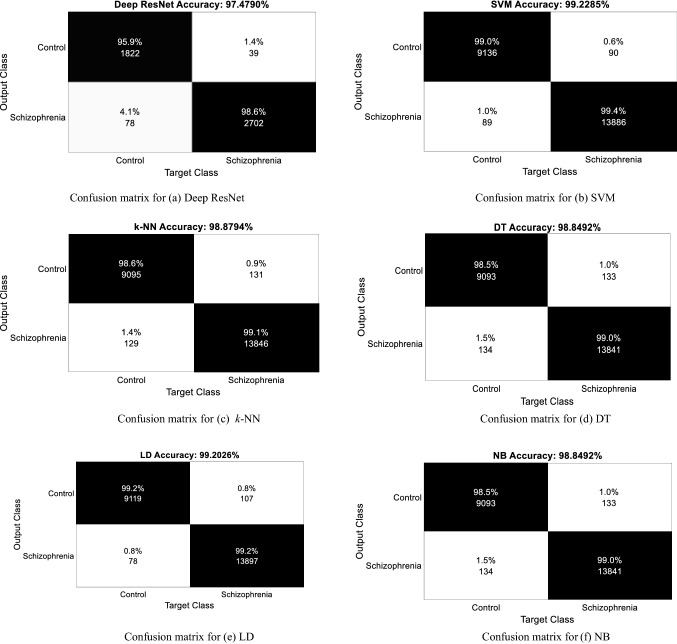
Confusion matrix for **a** Deep ResNet, **b** SVM, **c** *k*-NN, **d** DT, **e** LD, **f** NB

In order to show more detailed performance evidence such as class-specific performance, Fig. 8 presents a confusion matrix for each of the reported classification methods for the tenfold cross-validation procedure. These figures clearly display each label of data in the testing sets are predicted. The total number of each row represents the number of data predicted for that category while each column represents the true attribution of the category of the data. As seen in Fig. 8a, 2702 (98.60%) schizophrenia class data points are correctly identified by the deep ResNet classification model and 1822 (95.90%) normal control class data points are correctly classified by the model. On the other hand, 39 (1.4%) normal control data points are incorrectly identified as schizophrenia, and 78 (4.1%) schizophrenia data points are misclassified as normal control by the deep ResNet classifier. Similarly, the class-specific performance information for other classification methods such as for the SVM, *k*-NN, DT, LD, and NB are seen in Fig. 8b–f, respectively.

**Fig. 9:**
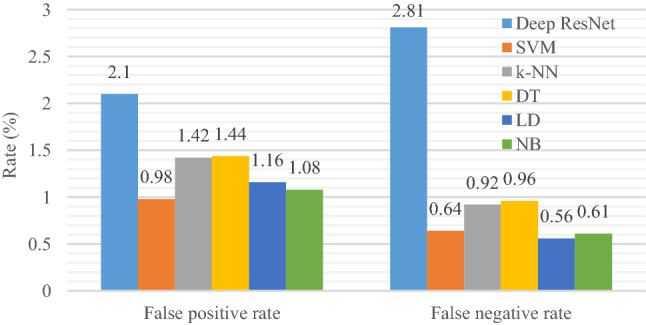
Comparison of false positive rate (false alarm rate) and false negative rate (miss rate) among the reported classifiers

To provide further information about the classification performance, a false positive rate, and false negative rate for the proposed models are illustrated in Fig. 9. The lower false positive rate and false negative rate indicate the better quality of a classification method. It is seen from this figure that the lowest false positive rate (0.98%) is obtained by the SVM classifier, while the LD produces the lowest false negative rate (0.56%). The highest false positive rate (2.1%) and the highest false negative rate (2.81%) is attained by the deep ResNet classifier (DL technique). In both evaluation parameters, the deep features with the SVM classifier (ML technique) perform better compared to the DL method like the previous performance measurement.

**Fig. 10:**
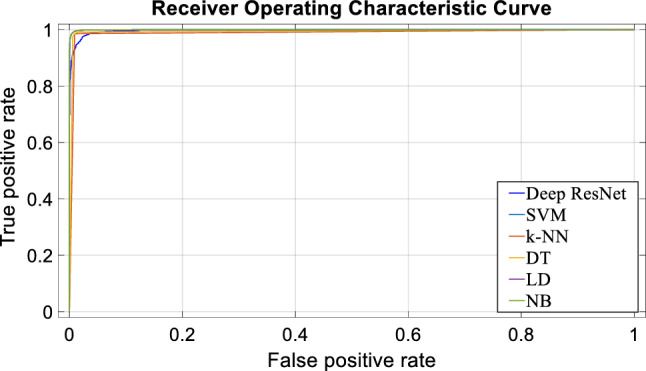
Performance comparison of the proposed classifiers by the ROC curve

**Fig. 11:**
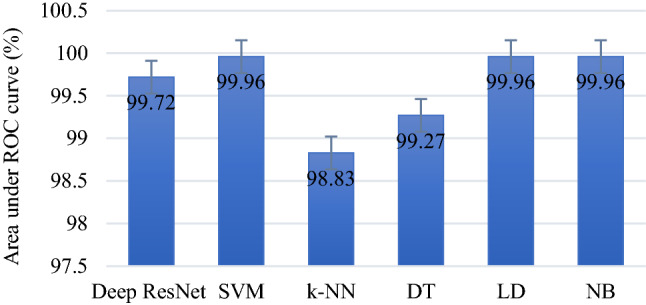
Area values of ROC curves for the considered classification methods

To more validate the efficacy of the proposed model, Fig. 10 presents a ROC curve comparing the performance of the deep ResNet, SVM, *k*-NN, DT, LD, and NB classifiers for the same the deep feature set. The ROC curve is drawn putting true positive rates (sensitivity) in X axis and false-positive rates in Y axis. An overall performance of a classifier is measured through the area value under the ROC curve which belongs to between 0 and 1 (bigger area value reveals better performance of the classifier). Figure 11 displays the area values under the curve (AUC) for the reported classifiers. In Fig. 11, vertical lines on the top of the bar charts show standard error among the classifiers. As can be seen in Figs. 10 and 11, the highest AUC is obtained by three ML classifiers: SVM, LD and NB which is 99.96% (close to 100%). The *k*-NN yields the lowest AUC for the same feature set.

To assess the computational complexity of the proposed models, this study compares the execution (running) time for the various classification methods in the training part and testing part. It is seen from Table 5 that in both training and testing part, the deep ResNet (DL classifier) took more time compared to the reported ML classifiers. The highest training time (14,749 s) and the highest testing time (20.50 s) were obtained by the deep ResNet classifier. The LD classifier took the lowest time (0.5310 s) in the training part and the NB took the lowest time (0.01563 s) in the testing part. The SVM classifier took the second lowest time (0.0313 s) in the testing part. It is worthy to mention that the time of ResNet model includes feature extraction and also classification process. The ML methods consider only classification process time.

**Table 5:** Comparision of computational complexcity of the proposed classifiers

Classification method	Training time (second)	Testing time (second)
Deep ResNet	14,749.00	20.50
SVM	32.1410	0.0313
KNN	0.7190	0.2031
DT	0.6090	0.1250
LD	0.5310	0.0625
NB	0.703	0.01563

In this study, in most of the circumstances, the proposed deep features perform better with ML classifiers compared to the DL classifier. Specially, the deep feature set with the SVM algorithm produced higher performance compared to other reported classifiers. The high classification performance across the SVM algorithm proves the deep features are highly discriminating between the two classes: schizophrenia and normal control. Thus, we can argue strongly that the deep features obtained from the ResNet model are perfect represent of EEG signals and the SVM classifier is the best choice for detecting schizophrenia category EEG signals from normal control signals.

### Comparison with the existing methods for the same dataset

**Fig.12:**
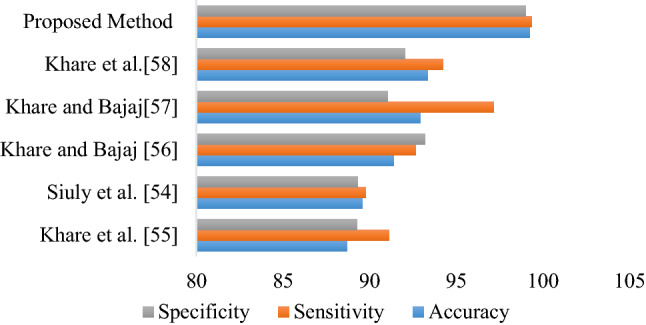
Comparison of the proposed scheme with other methods for the same database

This section provides a comparative report for our proposed method with the existing methods for the same Kaggle schizophrenia EEG dataset that was used in this study. Figure 12 presents the comparative report including the overall accuracy of the proposed method and the existing methods. Siuly et al. [54] developed an empirical mode decomposition-based features with ensemble bagged tree for the detection of schizophrenia from EEG signals. They obtained accuracy, sensitivity and specificity 89.59%, 89.76% and 89.32%, respectively. Khare et al. [55] introduced a method based on empirical wavelet transformation and SVM for the detection of schizophrenia from EEG signals. Their method achieved an accuracy of 88.70%, sensitivity of 91.13% and specificity of 89.29. For the EEG-based schizophrenia identification, Khare et al. [56] developed a flexible tuneable Q wavelet transform (F-TQWT) based methodology with least square support vector machine (F-LSSVM) that achieved 91.39% accuracy, 92.65% sensitivity, and 93.22% specificity. An optimised extreme learning machine (OELM) algorithm with a resilient variational mode decomposition (RVMD) foundation was proposed by Khare et al. in [57] and achieved an overall accuracy of 92.30%. In [58], Khare et al. proposed a convolutional neural network (CNN) model based on time–frequency analysis technique for detecting schizophrenia in EEG signals and obtained an overall accuracy of 93.36%. It is seen from Fig. 12 that our proposed model yielded the highest performance scores with the accuracy of 99.23%; sensitivity of 99.36% and specificity of 99.02%, compared to the existing two methods. The achieved accuracy improvement of our proposed model is 10.53% better than Khare et al. [55] accuracy score and 9.47% better than Siuly et al. [54] accuracy score. Towards the end, it can be stated that deep ResNet features of EEG signals with SVM classifier could be worked as an appropriate measurement to correctly distinguish schizophrenics and HC subjects.

## Conclusions

In this study, a deep ResNet model-based DL framework is proposed to learn robust feature representations of EEG signals for automatic detection of schizophrenia detection from normal control subjects, improving the performances. The proposed network architecture is designed combining convolutional, residual connections, average pooling, and fully connected layers that provide good convergence with higher performance. This proposed model can automatically extract effective features which are advantageous for large scale data. In this study, we extracted deep features using the deep ResNet model and then used as input to the softmax function for classification. To examine the performance of the obtained deep features, we also used the same feature set to five ML methods: SVM, *k*-NN, DT, LD, and NB, separately. The performance of the proposed method was evaluated on a benchmark schizophrenia EEG dataset from Kaggle. The extensive experiments were conducted, and the achieved results reveal that the SVM classifier with the acquired deep feature set produces the highest accuracy of 99.23% compared to the reported classifiers while this value is 97.48% for the softmax classifier of the ResNet model. Also, the SVM model with the deep feature set produces the lowest false positive rate (0.98%) where this value is 2.1% for the deep ResNet model. Moreover, we also compute the computational complexity of the proposed models. It is seen that the DL based model took a very long time (14,749.00 s) for the training part and testing part (20.50 s) compared to the reported ML-based methods. The findings of this study indicate that deep feature performs better with the ML classifier in the schizophrenia detection compared to the DL classification method. The results prove that the proposed method has the capability to discover hidden important biomarkers from EEG for automatic detection of schizophrenia that can assist technologists to build up a software system.

## Data Availability

This study used Kaggle data set: "EEG data from basic sensory task in Schizophrenia", which is publicly available in the below link: https://www.kaggle.com/datasets/broach/button-tone-sz To get this dataset, first need to be register in Kaggle, then dataset will be accessible
